# 4,4′-Bipyridinium bis(perchlorate)–4-aminobenzoic acid–4,4′-bipyridine–water (1/4/2/2)

**DOI:** 10.1107/S1600536808042207

**Published:** 2008-12-17

**Authors:** Qun-Hui Meng, Lu Han, Jian-Dong Hou, Yi-Fan Luo, Rong-Hua Zeng

**Affiliations:** a School of Chemistry and the Environment, South China Normal University, Guangzhou 510006, People’s Republic of China; b Key Laboratory of Technology on Electrochemical Energy Storage and Power Generation in Guangdong Universities, Guangzhou 510631, People’s Republic of China

## Abstract

In the structure of the title compound, C_10_H_10_N_2_^2+^·2ClO_4_^−^·4C_7_H_7_NO_2_·2C_10_H_8_N_2_·2H_2_O, the 4,4′-bipyridinium cation has a crystallographically imposed centre of symmetry. The cation is linked by N—H⋯N hydrogen bonds to adjacent 4,4′-bipyridine mol­ecules, which in turn inter­act *via* O—H⋯N hydrogen bonds with 4-amino­benzoic acid mol­ecules, forming chains running parallel to [30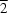]. The chains are further connected into a three-dimensional network by N—H⋯O and O—H⋯O hydrogen-bonding inter­actions involving the perchlorate anion, the water mol­ecules and the 4-amino­benzoic acid mol­ecules. In addition, π–π stacking inter­actions with centroid–centroid distances ranging from 3.663 (6) to 3.695 (6) Å are present. The O atoms of the perchlorate anion are disordered over two sets of positions, with refined site occupancies of 0.724 (9) and 0.276 (9).

## Related literature

For details of the hydrogen-bonding networks formed by 4-amino­benzoic acid and 4,4-bipyridine, see: Clemente & Marzotto (2004 ▶); Hu *et al.* (2003 ▶); Yang *et al.* (2004 ▶).
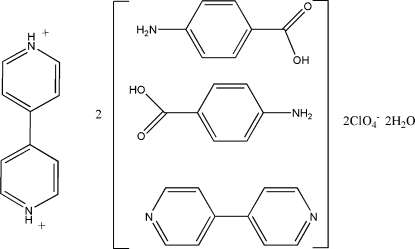


## Experimental

### 

#### Crystal data

C_10_H_10_N_2_^2+^·2ClO_4_^−^·4C_7_H_7_NO_2_·2C_10_H_8_N_2_·2H_2_O
*M* *_r_* = 1254.04
Triclinic, 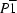

*a* = 10.1007 (3) Å
*b* = 10.1105 (4) Å
*c* = 16.5830 (6) Å
α = 91.487 (2)°
β = 98.529 (3)°
γ = 118.500 (2)°
*V* = 1462.68 (10) Å^3^
*Z* = 1
Mo *K*α radiation
μ = 0.19 mm^−1^
*T* = 296 (2) K
0.20 × 0.18 × 0.15 mm

#### Data collection

Bruker APEXII area-detector diffractometer
Absorption correction: none
18132 measured reflections
5242 independent reflections
3142 reflections with *I* > 2σ(*I*)
*R*_int_ = 0.058

#### Refinement

*R*[*F* ^2^ > 2σ(*F* ^2^)] = 0.066
*wR*(*F* ^2^) = 0.198
*S* = 1.04
5242 reflections
455 parameters
74 restraints
H atoms treated by a mixture of independent and constrained refinement
Δρ_max_ = 0.37 e Å^−3^
Δρ_min_ = −0.41 e Å^−3^

### 

Data collection: *APEX2* (Bruker, 2004 ▶); cell refinement: *SAINT* (Bruker, 2004 ▶); data reduction: *SAINT*; program(s) used to solve structure: *SHELXS97* (Sheldrick, 2008 ▶); program(s) used to refine structure: *SHELXL97* (Sheldrick, 2008 ▶); molecular graphics: *XP* in *SHELXTL* (Sheldrick, 2008 ▶); software used to prepare material for publication: *SHELXL97*.

## Supplementary Material

Crystal structure: contains datablocks I, global. DOI: 10.1107/S1600536808042207/rz2274sup1.cif

Structure factors: contains datablocks I. DOI: 10.1107/S1600536808042207/rz2274Isup2.hkl

Additional supplementary materials:  crystallographic information; 3D view; checkCIF report

## Figures and Tables

**Table 1:** Hydrogen-bond geometry (Å, °)

*D*—H⋯*A*	*D*—H	H⋯*A*	*D*⋯*A*	*D*—H⋯*A*
O1—H1*C*⋯N2^i^	0.89 (4)	1.78 (4)	2.673 (4)	174 (5)
O3—H3*C*⋯O5^ii^	0.90 (5)	1.72 (4)	2.609 (4)	168 (5)
O5—H5*B*⋯O4^iii^	0.86 (4)	1.93 (4)	2.775 (4)	171 (4)
O5—H5*A*⋯O2^iv^	0.852 (11)	1.903 (13)	2.739 (4)	167 (3)
N5—H5*C*⋯N1	0.90 (5)	1.78 (5)	2.680 (4)	175 (5)
N3—H3*A*⋯O6	0.86	2.24	3.079 (6)	166
N3—H3*A*⋯O7′	0.86	2.31	3.113 (15)	156
N3—H3*B*⋯O6^v^	0.86	2.25	3.085 (8)	166
N3—H3*B*⋯O6′^v^	0.86	2.09	2.917 (18)	162
N4—H4*A*⋯O8^vi^	0.86	2.22	3.028 (10)	157
N4—H4*A*⋯O8′^vi^	0.86	2.55	3.351 (19)	156
N4—H4*B*⋯O8	0.86	2.57	3.174 (13)	129
N4—H4*B*⋯O8′	0.86	2.57	3.25 (2)	136

Symmetry codes: (i) 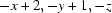; (ii) 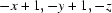; (iii) 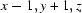; (iv) 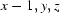; (v) 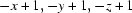; (vi) 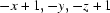.

**Fig. 1.**
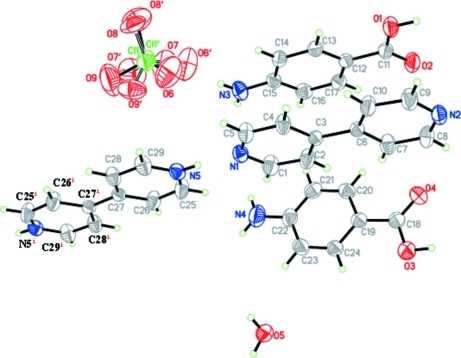
The molecular structure showing the atomic-numbering scheme. Displacement ellipsoids drawn at the 30% probability level. Symmetry codes: (i)-x, 1-y, 1-z.

**Fig. 2.**
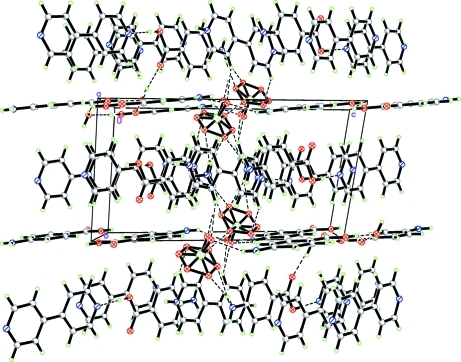
Packing diagram of the title compound viewed along the a axis. Hydrogen bonds are shown as dashed lines.

## supplementary crystallographic information

### Comment

Hydrogen-bonding interactions between ligands are specific and directional. In this sense, 4-aminobenzoic acid and 4,4-bipyridine are the excellent candidates for the construction of three-dimensional network motifs, as they form regular hydrogen bonds functioning as both hydrogen-bond donors and acceptors (Clemente & Marzotto, 2004; Hu *et al.*, 2003; Yang *et al.*, 2004). Recently, we obtained the title compound under hydrothermal conditions and report its crystal structure herein.

In the title compound (Fig. 1), all bond lengths and angles are unexceptional. The 4,4'-bipyridinium cation, which is located on a centre of symmetry, acts as a N—H hydrogen bond donor on both sides to unprotonated 4,4'-bipyridine molecules, which in turn act as O—H hydrogen bond acceptors from one of the two independent 4-aminobenzoic acid molecules. Two 4-aminobenzoic acid molecules, two 4,4'-bipyridine molecules and the cation are thus connected by hydrogen bonding interactions to form a linear centrosymmetric chain running parallel to the [3 0 2] direction. These chains are further connected by O—H···O and N—H···O hydrogen bonds (Table 1) *via* the interstitial solvate water molecules, the perchlorate anions and the other 4-aminobenzoic acid molecules, forming a three-dimensional network (Fig. 2). The crystal packing is stabilized by π-π stacking interactions involving both pyridine rings of the unprotonated 4,4'-bipyridine molecule, the N5/C25-C29 pyridine ring of the cation and the C12-C17 benzene ring of one 4-aminobenzoic acid molecule: *Cg1*···*Cg2^i^* = 3.695 (6) Å; *Cg1*···*Cg3* = 3.663 (6) Å; *Cg3*···*Cg4^ii^* = 3.674 (7) Å [*Cg1*, *Cg2*, *Cg3* and *Cg4* are the centroids of the N1/C1–C5, N2/C6–C10, C12–CC17 and N5/C25–C29 rings, respectively. Symmetry codes: (i) = 1-x, 1-y, -z; (ii) = 1-x, y, z].

### Experimental

4-Aminobenzoic acid (1 mmol, 0.137 g,), 4,4-bipyridine (1 mmol, 0.156 g,) and sodium perchlorate (1 mmol, 0.123 g,) were dissolved in hot water with stirring. Colourless single crystals were obtained at room temperature by slow evaporation of the solvent over a period of several days.

### Refinement

The disordered perchlorate ion was refined into two sites with refined occupancies of 0.724 (9) and 0.276 (9). The Cl···O and O···O distances were restrained to be 1.44 (1) and 2.35 (1) Å, respectively. Water H atoms were located in a difference Fourier map and refined with distance restraints of O–H = 0.86 Å and H···H = 1.39 Å. The H atom bound to the N5 nitrogen atom in the cation and the carboxylic H atoms were refined with N—H and O—H distance restraints of 0.90 Å. All other H atoms were placed at calculated positions and treated as riding on the parent atoms, with C—H = 0.93\ %A, N—H = 0.86 Å, and with *U*_iso_(H) = 1.2 *U*_eq_(C, N).

### Crystal data

**Table:**

C_10_H_10_N_2_^2+^·2ClO_4_^−^·4C_7_H_7_NO_2_·2C_10_H_8_N_2_·2H_2_O	*Z* = 1
*M**_r_* = 1254.04	*F*(000) = 654
Triclinic, *P*1	*D*_x_ = 1.424 Mg m^−^^3^
Hall symbol: -P 1	Mo *K*α radiation, λ = 0.71073 Å
*a* = 10.1007 (3) Å	Cell parameters from 2787 reflections
*b* = 10.1105 (4) Å	θ = 2.4–21.0°
*c* = 16.5830 (6) Å	µ = 0.19 mm^−^^1^
α = 91.487 (2)°	*T* = 296 K
β = 98.529 (3)°	Block, colourless
γ = 118.500 (2)°	0.20 × 0.18 × 0.15 mm
*V* = 1462.68 (10) Å^3^	

### Data collection

**Table:**

Bruker APEXII area-detector diffractometer	3142 reflections with *I* > 2σ(*I*)
Radiation source: fine-focus sealed tube	*R*_int_ = 0.058
graphite	θ_max_ = 25.2°, θ_min_ = 2.3°
φ and ω scans	*h* = −12→11
18132 measured reflections	*k* = −11→12
5242 independent reflections	*l* = −18→19

### Refinement

**Table:**

Refinement on *F*^2^	Primary atom site location: structure-invariant direct methods
Least-squares matrix: full	Secondary atom site location: difference Fourier map
*R*[*F*^2^ > 2σ(*F*^2^)] = 0.066	Hydrogen site location: inferred from neighbouring sites
*wR*(*F*^2^) = 0.198	H atoms treated by a mixture of independent and constrained refinement
*S* = 1.04	*w* = 1/[σ^2^(*F*_o_^2^) + (0.0835*P*)^2^ + 0.5543*P*] where *P* = (*F*_o_^2^ + 2*F*_c_^2^)/3
5242 reflections	(Δ/σ)_max_ < 0.001
455 parameters	Δρ_max_ = 0.37 e Å^−^^3^
74 restraints	Δρ_min_ = −0.41 e Å^−^^3^

### Special details

**Table:**

Geometry. All esds (except the esd in the dihedral angle between two l.s. planes) are estimated using the full covariance matrix. The cell esds are taken into account individually in the estimation of esds in distances, angles and torsion angles; correlations between esds in cell parameters are only used when they are defined by crystal symmetry. An approximate (isotropic) treatment of cell esds is used for estimating esds involving l.s. planes.
Refinement. Refinement of F^2^ against ALL reflections. The weighted R-factor wR and goodness of fit S are based on F^2^, conventional R-factors R are based on F, with F set to zero for negative F^2^. The threshold expression of F^2^ > 2sigma(F^2^) is used only for calculating R-factors(gt) etc. and is not relevant to the choice of reflections for refinement. R-factors based on F^2^ are statistically about twice as large as those based on F, and R- factors based on ALL data will be even larger.

### Fractional atomic coordinates and isotropic or equivalent isotropic displacement parameters (Å^2^)

**Table:**

	*x*	*y*	*z*	*U*_iso_*/*U*_eq_	Occ. (<1)
C1	0.4592 (5)	0.5702 (4)	0.2272 (2)	0.0780 (12)	
H1	0.4688	0.6561	0.2552	0.094*	
C2	0.5531 (5)	0.5894 (4)	0.1716 (2)	0.0718 (11)	
H2	0.6235	0.6866	0.1627	0.086*	
C3	0.5438 (3)	0.4659 (3)	0.12897 (17)	0.0445 (7)	
C4	0.4368 (4)	0.3262 (4)	0.1468 (2)	0.0621 (9)	
H4	0.4256	0.2382	0.1204	0.075*	
C5	0.3468 (4)	0.3170 (4)	0.2035 (2)	0.0667 (10)	
H5	0.2758	0.2216	0.2145	0.080*	
C6	0.6445 (3)	0.4829 (3)	0.06907 (17)	0.0454 (7)	
C7	0.7468 (4)	0.6237 (4)	0.0504 (2)	0.0640 (10)	
H7	0.7552	0.7104	0.0767	0.077*	
C8	0.8361 (4)	0.6363 (4)	−0.0067 (2)	0.0681 (10)	
H8	0.9044	0.7329	−0.0176	0.082*	
C9	0.7333 (5)	0.3836 (5)	−0.0301 (2)	0.0774 (12)	
H9	0.7272	0.2990	−0.0576	0.093*	
C10	0.6398 (5)	0.3613 (4)	0.0269 (2)	0.0716 (11)	
H10	0.5731	0.2634	0.0368	0.086*	
C11	1.0179 (4)	0.5687 (4)	0.1962 (2)	0.0570 (9)	
C12	0.9160 (4)	0.5534 (4)	0.25473 (18)	0.0504 (8)	
C13	0.8008 (4)	0.4134 (4)	0.26858 (19)	0.0529 (8)	
H13	0.7875	0.3258	0.2406	0.064*	
C14	0.7066 (4)	0.4019 (4)	0.32255 (19)	0.0556 (8)	
H14	0.6304	0.3070	0.3307	0.067*	
C15	0.7238 (4)	0.5309 (4)	0.3653 (2)	0.0566 (9)	
C16	0.8404 (4)	0.6714 (4)	0.3531 (2)	0.0647 (10)	
H16	0.8550	0.7588	0.3820	0.078*	
C17	0.9340 (4)	0.6819 (4)	0.2984 (2)	0.0601 (9)	
H17	1.0108	0.7767	0.2904	0.072*	
C18	0.8014 (5)	0.0246 (4)	0.0816 (2)	0.0625 (9)	
C19	0.7278 (4)	0.0094 (3)	0.1525 (2)	0.0573 (9)	
C20	0.8008 (4)	0.0010 (4)	0.2290 (2)	0.0653 (10)	
H20	0.8963	0.0066	0.2340	0.078*	
C21	0.7339 (5)	−0.0152 (4)	0.2969 (2)	0.0713 (11)	
H21	0.7847	−0.0200	0.3473	0.086*	
C22	0.5911 (5)	−0.0244 (4)	0.2912 (3)	0.0735 (11)	
C23	0.5181 (5)	−0.0161 (4)	0.2145 (3)	0.0747 (11)	
H23	0.4226	−0.0216	0.2094	0.090*	
C24	0.5850 (4)	0.0000 (4)	0.1471 (2)	0.0644 (10)	
H24	0.5341	0.0047	0.0966	0.077*	
C25	0.2424 (5)	0.5900 (5)	0.3916 (2)	0.0785 (12)	
H25	0.3251	0.6733	0.3770	0.094*	
C26	0.1654 (5)	0.6129 (4)	0.4471 (2)	0.0716 (11)	
H26	0.1951	0.7107	0.4689	0.086*	
C27	0.0433 (3)	0.4902 (4)	0.47059 (17)	0.0466 (7)	
C28	0.0067 (4)	0.3484 (4)	0.4352 (2)	0.0657 (10)	
H28	−0.0737	0.2620	0.4491	0.079*	
C29	0.0886 (4)	0.3356 (5)	0.3799 (2)	0.0709 (11)	
H29	0.0623	0.2396	0.3567	0.085*	
N1	0.3567 (3)	0.4374 (3)	0.24310 (16)	0.0579 (7)	
N2	0.8310 (3)	0.5191 (4)	−0.04737 (17)	0.0607 (8)	
N3	0.6272 (4)	0.5201 (4)	0.41820 (18)	0.0758 (9)	
H3A	0.5550	0.4328	0.4251	0.091*	
H3B	0.6389	0.6007	0.4443	0.091*	
N4	0.5226 (5)	−0.0444 (5)	0.3583 (2)	0.1091 (13)	
H4A	0.5680	−0.0514	0.4049	0.131*	
H4B	0.4342	−0.0499	0.3540	0.131*	
N5	0.2031 (4)	0.4537 (4)	0.35815 (17)	0.0598 (8)	
O1	0.9981 (3)	0.4406 (3)	0.16264 (16)	0.0718 (7)	
O2	1.1150 (3)	0.6913 (3)	0.18048 (17)	0.0787 (8)	
O3	0.7226 (3)	0.0324 (3)	0.01186 (18)	0.0793 (8)	
O4	0.9250 (3)	0.0302 (3)	0.08352 (18)	0.0848 (8)	
O5	0.1553 (3)	0.9533 (3)	0.11791 (17)	0.0720 (7)	
H1C	1.058 (4)	0.451 (5)	0.126 (2)	0.119 (17)*	
H3C	0.776 (5)	0.039 (6)	−0.028 (2)	0.122 (19)*	
H5A	0.128 (4)	0.866 (2)	0.133 (2)	0.078 (13)*	
H5B	0.079 (3)	0.970 (4)	0.111 (3)	0.12 (2)*	
H5C	0.251 (5)	0.442 (6)	0.319 (2)	0.14 (2)*	
O6	0.3808 (7)	0.2312 (5)	0.4742 (4)	0.153 (3)	0.724 (9)
O7	0.2540 (10)	0.0812 (8)	0.3516 (3)	0.158 (3)	0.724 (9)
O8	0.2998 (12)	−0.0234 (8)	0.4681 (6)	0.155 (4)	0.724 (9)
O9	0.1258 (8)	0.0621 (9)	0.4610 (6)	0.190 (4)	0.724 (9)
Cl1	0.26368 (13)	0.08898 (12)	0.43474 (8)	0.0877 (4)	0.724 (9)
O6'	0.265 (2)	0.1766 (19)	0.5022 (7)	0.158 (7)	0.276 (9)
O7'	0.3803 (16)	0.1791 (19)	0.3902 (11)	0.175 (8)	0.276 (9)
O8'	0.264 (2)	−0.0437 (14)	0.4533 (12)	0.109 (7)	0.276 (9)
O9'	0.1148 (13)	0.0416 (13)	0.3755 (9)	0.115 (6)	0.276 (9)
Cl1'	0.26368 (13)	0.08898 (12)	0.43474 (8)	0.0877 (4)	0.276 (9)

### Atomic displacement parameters (Å^2^)

**Table:**

	*U*^11^	*U*^22^	*U*^33^	*U*^12^	*U*^13^	*U*^23^
C1	0.101 (3)	0.059 (2)	0.088 (3)	0.041 (2)	0.048 (2)	0.007 (2)
C2	0.087 (3)	0.048 (2)	0.086 (3)	0.029 (2)	0.045 (2)	0.0136 (19)
C3	0.0441 (17)	0.0503 (19)	0.0398 (16)	0.0236 (15)	0.0071 (13)	0.0044 (13)
C4	0.068 (2)	0.050 (2)	0.067 (2)	0.0218 (18)	0.0303 (18)	0.0017 (16)
C5	0.065 (2)	0.057 (2)	0.071 (2)	0.0190 (18)	0.0278 (18)	0.0058 (18)
C6	0.0450 (17)	0.0509 (19)	0.0415 (16)	0.0240 (15)	0.0085 (13)	0.0076 (14)
C7	0.062 (2)	0.051 (2)	0.070 (2)	0.0167 (17)	0.0244 (18)	0.0000 (17)
C8	0.057 (2)	0.062 (2)	0.068 (2)	0.0113 (18)	0.0246 (18)	0.0100 (19)
C9	0.099 (3)	0.066 (3)	0.084 (3)	0.043 (2)	0.051 (2)	0.014 (2)
C10	0.088 (3)	0.051 (2)	0.086 (3)	0.033 (2)	0.050 (2)	0.0181 (19)
C11	0.051 (2)	0.067 (2)	0.053 (2)	0.0286 (19)	0.0115 (16)	0.0133 (18)
C12	0.0505 (19)	0.058 (2)	0.0458 (17)	0.0287 (17)	0.0082 (14)	0.0096 (15)
C13	0.057 (2)	0.055 (2)	0.0496 (18)	0.0293 (17)	0.0115 (15)	0.0056 (15)
C14	0.054 (2)	0.059 (2)	0.0524 (19)	0.0248 (17)	0.0139 (15)	0.0097 (16)
C15	0.056 (2)	0.074 (2)	0.0492 (19)	0.0380 (19)	0.0113 (16)	0.0105 (17)
C16	0.073 (2)	0.063 (2)	0.066 (2)	0.039 (2)	0.0136 (19)	0.0012 (18)
C17	0.059 (2)	0.054 (2)	0.065 (2)	0.0247 (17)	0.0153 (17)	0.0110 (17)
C18	0.066 (2)	0.047 (2)	0.077 (3)	0.0274 (18)	0.018 (2)	0.0114 (17)
C19	0.056 (2)	0.0402 (18)	0.074 (2)	0.0217 (16)	0.0126 (17)	0.0086 (16)
C20	0.062 (2)	0.057 (2)	0.077 (3)	0.0304 (19)	0.0102 (19)	0.0050 (18)
C21	0.084 (3)	0.059 (2)	0.069 (2)	0.034 (2)	0.011 (2)	0.0068 (18)
C22	0.082 (3)	0.050 (2)	0.089 (3)	0.027 (2)	0.034 (2)	0.014 (2)
C23	0.061 (2)	0.064 (2)	0.103 (3)	0.031 (2)	0.023 (2)	0.019 (2)
C24	0.061 (2)	0.056 (2)	0.079 (3)	0.0292 (18)	0.0149 (19)	0.0149 (18)
C25	0.090 (3)	0.067 (3)	0.083 (3)	0.032 (2)	0.050 (2)	0.017 (2)
C26	0.088 (3)	0.053 (2)	0.083 (3)	0.033 (2)	0.047 (2)	0.0095 (18)
C27	0.0489 (18)	0.0546 (19)	0.0420 (16)	0.0294 (16)	0.0090 (13)	0.0066 (14)
C28	0.060 (2)	0.056 (2)	0.078 (2)	0.0229 (18)	0.0246 (18)	−0.0046 (18)
C29	0.066 (2)	0.063 (2)	0.077 (3)	0.026 (2)	0.021 (2)	−0.0122 (19)
N1	0.0571 (17)	0.067 (2)	0.0516 (16)	0.0300 (15)	0.0159 (13)	0.0041 (14)
N2	0.0552 (17)	0.074 (2)	0.0550 (17)	0.0302 (16)	0.0203 (13)	0.0093 (15)
N3	0.073 (2)	0.093 (2)	0.072 (2)	0.0439 (19)	0.0284 (17)	0.0050 (18)
N4	0.125 (3)	0.118 (3)	0.103 (3)	0.062 (3)	0.060 (3)	0.037 (3)
N5	0.0637 (19)	0.077 (2)	0.0497 (16)	0.0412 (18)	0.0156 (14)	0.0034 (15)
O1	0.0788 (18)	0.0675 (17)	0.0739 (17)	0.0323 (14)	0.0395 (14)	0.0093 (14)
O2	0.0747 (17)	0.0691 (17)	0.0948 (19)	0.0289 (15)	0.0418 (15)	0.0247 (15)
O3	0.0734 (18)	0.095 (2)	0.0739 (19)	0.0426 (16)	0.0169 (15)	0.0200 (15)
O4	0.0781 (19)	0.101 (2)	0.098 (2)	0.0563 (17)	0.0312 (16)	0.0246 (16)
O5	0.0724 (18)	0.0648 (18)	0.0782 (18)	0.0317 (15)	0.0163 (14)	0.0169 (14)
O6	0.165 (5)	0.067 (3)	0.187 (6)	0.018 (3)	0.060 (4)	−0.015 (3)
O7	0.213 (8)	0.156 (5)	0.086 (4)	0.072 (5)	0.043 (4)	0.021 (3)
O8	0.199 (8)	0.095 (5)	0.177 (7)	0.077 (5)	0.030 (6)	0.017 (4)
O9	0.148 (6)	0.210 (7)	0.237 (8)	0.084 (5)	0.114 (6)	0.047 (6)
Cl1	0.0960 (9)	0.0596 (7)	0.1150 (10)	0.0368 (6)	0.0438 (7)	0.0144 (6)
O6'	0.195 (12)	0.143 (10)	0.156 (10)	0.094 (9)	0.056 (8)	−0.036 (7)
O7'	0.172 (11)	0.162 (11)	0.170 (12)	0.048 (8)	0.082 (9)	0.058 (9)
O8'	0.115 (9)	0.063 (9)	0.145 (11)	0.045 (7)	−0.003 (7)	0.037 (7)
O9'	0.095 (8)	0.096 (8)	0.139 (10)	0.051 (6)	−0.030 (7)	−0.010 (6)
Cl1'	0.0960 (9)	0.0596 (7)	0.1150 (10)	0.0368 (6)	0.0438 (7)	0.0144 (6)

### Geometric parameters (Å, °)

**Table:**

C1—N1	1.310 (5)	C18—C19	1.456 (5)
C1—C2	1.371 (5)	C19—C24	1.388 (5)
C1—H1	0.9300	C19—C20	1.396 (5)
C2—C3	1.376 (5)	C20—C21	1.372 (5)
C2—H2	0.9300	C20—H20	0.9300
C3—C4	1.382 (4)	C21—C22	1.388 (6)
C3—C6	1.481 (4)	C21—H21	0.9300
C4—C5	1.378 (5)	C22—N4	1.366 (5)
C4—H4	0.9300	C22—C23	1.397 (6)
C5—N1	1.321 (4)	C23—C24	1.365 (5)
C5—H5	0.9300	C23—H23	0.9300
C6—C10	1.375 (5)	C24—H24	0.9300
C6—C7	1.380 (4)	C25—N5	1.318 (5)
C7—C8	1.371 (5)	C25—C26	1.371 (5)
C7—H7	0.9300	C25—H25	0.9300
C8—N2	1.323 (5)	C26—C27	1.385 (5)
C8—H8	0.9300	C26—H26	0.9300
C9—N2	1.322 (5)	C27—C28	1.387 (5)
C9—C10	1.381 (5)	C27—C27^i^	1.472 (6)
C9—H9	0.9300	C28—C29	1.365 (5)
C10—H10	0.9300	C28—H28	0.9300
C11—O2	1.224 (4)	C29—N5	1.312 (5)
C11—O1	1.308 (4)	C29—H29	0.9300
C11—C12	1.475 (5)	N3—H3A	0.8600
C12—C17	1.390 (5)	N3—H3B	0.8600
C12—C13	1.391 (5)	N4—H4A	0.8600
C13—C14	1.368 (4)	N4—H4B	0.8600
C13—H13	0.9300	N5—H5C	0.90 (5)
C14—C15	1.390 (5)	O1—H1C	0.89 (4)
C14—H14	0.9300	O3—H3C	0.90 (5)
C15—N3	1.375 (4)	O5—H5A	0.852 (11)
C15—C16	1.391 (5)	O5—H5B	0.86 (4)
C16—C17	1.376 (5)	O6—Cl1	1.412 (4)
C16—H16	0.9300	O7—Cl1	1.365 (4)
C17—H17	0.9300	O8—Cl1	1.448 (5)
C18—O4	1.218 (4)	O9—Cl1	1.424 (5)
C18—O3	1.329 (4)		
			
N1—C1—C2	123.6 (3)	C24—C19—C20	118.0 (3)
N1—C1—H1	118.2	C24—C19—C18	122.5 (3)
C2—C1—H1	118.2	C20—C19—C18	119.5 (3)
C1—C2—C3	120.4 (3)	C21—C20—C19	121.0 (4)
C1—C2—H2	119.8	C21—C20—H20	119.5
C3—C2—H2	119.8	C19—C20—H20	119.5
C2—C3—C4	115.7 (3)	C20—C21—C22	120.8 (4)
C2—C3—C6	121.7 (3)	C20—C21—H21	119.6
C4—C3—C6	122.6 (3)	C22—C21—H21	119.6
C5—C4—C3	120.2 (3)	N4—C22—C21	120.8 (4)
C5—C4—H4	119.9	N4—C22—C23	121.0 (4)
C3—C4—H4	119.9	C21—C22—C23	118.2 (4)
N1—C5—C4	123.0 (3)	C24—C23—C22	120.9 (4)
N1—C5—H5	118.5	C24—C23—H23	119.5
C4—C5—H5	118.5	C22—C23—H23	119.5
C10—C6—C7	115.7 (3)	C23—C24—C19	121.1 (4)
C10—C6—C3	122.9 (3)	C23—C24—H24	119.4
C7—C6—C3	121.4 (3)	C19—C24—H24	119.4
C8—C7—C6	120.3 (3)	N5—C25—C26	122.2 (4)
C8—C7—H7	119.8	N5—C25—H25	118.9
C6—C7—H7	119.8	C26—C25—H25	118.9
N2—C8—C7	123.9 (3)	C25—C26—C27	119.9 (3)
N2—C8—H8	118.1	C25—C26—H26	120.0
C7—C8—H8	118.1	C27—C26—H26	120.0
N2—C9—C10	123.3 (3)	C26—C27—C28	116.4 (3)
N2—C9—H9	118.4	C26—C27—C27^i^	121.7 (4)
C10—C9—H9	118.4	C28—C27—C27^i^	121.9 (4)
C6—C10—C9	120.5 (3)	C29—C28—C27	120.0 (3)
C6—C10—H10	119.8	C29—C28—H28	120.0
C9—C10—H10	119.8	C27—C28—H28	120.0
O2—C11—O1	122.0 (3)	N5—C29—C28	122.5 (4)
O2—C11—C12	123.2 (3)	N5—C29—H29	118.8
O1—C11—C12	114.8 (3)	C28—C29—H29	118.8
C17—C12—C13	117.9 (3)	C1—N1—C5	117.1 (3)
C17—C12—C11	119.8 (3)	C9—N2—C8	116.4 (3)
C13—C12—C11	122.3 (3)	C15—N3—H3A	120.0
C14—C13—C12	121.3 (3)	C15—N3—H3B	120.0
C14—C13—H13	119.3	H3A—N3—H3B	120.0
C12—C13—H13	119.3	C22—N4—H4A	120.0
C13—C14—C15	120.6 (3)	C22—N4—H4B	120.0
C13—C14—H14	119.7	H4A—N4—H4B	120.0
C15—C14—H14	119.7	C29—N5—C25	119.1 (3)
N3—C15—C14	120.8 (3)	C29—N5—H5C	120 (4)
N3—C15—C16	120.6 (3)	C25—N5—H5C	121 (4)
C14—C15—C16	118.6 (3)	C11—O1—H1C	114 (3)
C17—C16—C15	120.4 (3)	C18—O3—H3C	108 (3)
C17—C16—H16	119.8	H5A—O5—H5B	110 (4)
C15—C16—H16	119.8	O7—Cl1—O6	111.8 (4)
C16—C17—C12	121.2 (3)	O7—Cl1—O9	114.1 (5)
C16—C17—H17	119.4	O6—Cl1—O9	107.3 (4)
C12—C17—H17	119.4	O7—Cl1—O8	110.9 (4)
O4—C18—O3	120.9 (4)	O6—Cl1—O8	106.6 (5)
O4—C18—C19	124.5 (4)	O9—Cl1—O8	105.7 (5)
O3—C18—C19	114.6 (3)		
			
N1—C1—C2—C3	−0.3 (7)	C13—C12—C17—C16	−0.5 (5)
C1—C2—C3—C4	−0.5 (6)	C11—C12—C17—C16	179.7 (3)
C1—C2—C3—C6	−179.4 (4)	O4—C18—C19—C24	−178.5 (3)
C2—C3—C4—C5	0.6 (5)	O3—C18—C19—C24	1.6 (5)
C6—C3—C4—C5	179.5 (3)	O4—C18—C19—C20	0.3 (5)
C3—C4—C5—N1	0.1 (6)	O3—C18—C19—C20	−179.6 (3)
C2—C3—C6—C10	178.6 (3)	C24—C19—C20—C21	−0.3 (5)
C4—C3—C6—C10	−0.3 (5)	C18—C19—C20—C21	−179.2 (3)
C2—C3—C6—C7	−3.3 (5)	C19—C20—C21—C22	0.3 (5)
C4—C3—C6—C7	177.8 (3)	C20—C21—C22—N4	178.3 (4)
C10—C6—C7—C8	−0.2 (5)	C20—C21—C22—C23	−0.3 (5)
C3—C6—C7—C8	−178.4 (3)	N4—C22—C23—C24	−178.3 (4)
C6—C7—C8—N2	0.4 (6)	C21—C22—C23—C24	0.3 (6)
C7—C6—C10—C9	−0.1 (6)	C22—C23—C24—C19	−0.4 (6)
C3—C6—C10—C9	178.1 (3)	C20—C19—C24—C23	0.4 (5)
N2—C9—C10—C6	0.1 (7)	C18—C19—C24—C23	179.2 (3)
O2—C11—C12—C17	−3.6 (5)	N5—C25—C26—C27	1.0 (7)
O1—C11—C12—C17	175.5 (3)	C25—C26—C27—C28	0.0 (6)
O2—C11—C12—C13	176.5 (3)	C25—C26—C27—C27^i^	−179.4 (4)
O1—C11—C12—C13	−4.3 (5)	C26—C27—C28—C29	−0.5 (5)
C17—C12—C13—C14	0.8 (5)	C27^i^—C27—C28—C29	178.9 (4)
C11—C12—C13—C14	−179.3 (3)	C27—C28—C29—N5	0.1 (6)
C12—C13—C14—C15	−0.1 (5)	C2—C1—N1—C5	1.0 (6)
C13—C14—C15—N3	178.5 (3)	C4—C5—N1—C1	−0.9 (6)
C13—C14—C15—C16	−1.1 (5)	C10—C9—N2—C8	0.0 (6)
N3—C15—C16—C17	−178.2 (3)	C7—C8—N2—C9	−0.3 (6)
C14—C15—C16—C17	1.4 (5)	C28—C29—N5—C25	0.9 (6)
C15—C16—C17—C12	−0.6 (5)	C26—C25—N5—C29	−1.5 (6)

Symmetry codes: (i) −*x*, −*y*+1, −*z*+1.

### Hydrogen-bond geometry (Å, °)

**Table:**

*D*—H···*A*	*D*—H	H···*A*	*D*···*A*	*D*—H···*A*
O1—H1C···N2^ii^	0.89 (4)	1.78 (4)	2.673 (4)	174 (5)
O3—H3C···O5^iii^	0.90 (5)	1.72 (4)	2.609 (4)	168 (5)
O5—H5B···O4^iv^	0.86 (4)	1.93 (4)	2.775 (4)	171 (4)
O5—H5A···O2^v^	0.85 (1)	1.90 (1)	2.739 (4)	167 (3)
N5—H5C···N1	0.90 (5)	1.78 (5)	2.680 (4)	175 (5)
N3—H3A···O6	0.86	2.24	3.079 (6)	166
N3—H3A···O7'	0.86	2.31	3.113 (15)	156
N3—H3B···O6^vi^	0.86	2.25	3.085 (8)	166
N3—H3B···O6'^vi^	0.86	2.09	2.917 (18)	162
N4—H4A···O8^vii^	0.86	2.22	3.028 (10)	157
N4—H4A···O8'^vii^	0.86	2.55	3.351 (19)	156
N4—H4B···O8	0.86	2.57	3.174 (13)	129
N4—H4B···O8'	0.86	2.57	3.25 (2)	136

Symmetry codes: (ii) −*x*+2, −*y*+1, −*z*; (iii) −*x*+1, −*y*+1, −*z*; (iv) *x*−1, *y*+1, *z*; (v) *x*−1, *y*, *z*; (vi) −*x*+1, −*y*+1, −*z*+1; (vii) −*x*+1, −*y*, −*z*+1.
